# Chronic Exposure to Hypoxia Inhibits Myelinogenesis and Causes Motor Coordination Deficits in Adult Mice

**DOI:** 10.1007/s12264-021-00745-1

**Published:** 2021-07-22

**Authors:** Lin Chen, Shu-Yu Ren, Rui-Xue Li, Kun Liu, Jing-Fei Chen, Yu-Jian Yang, Yong-Bin Deng, Han-Zhi Wang, Lan Xiao, Feng Mei, Fei Wang

**Affiliations:** 1 Department of Histology and Embryology, Chongqing Key Laboratory of Neurobiology, Brain and Intelligence Research Key Laboratory of Chongqing Education Commission, Third Military Medical University, Chongqing, 400038 China; 2 Department of Neurosurgery, Chongqing Emergency Medical Center, Chongqing University, Chongqing, 400014 China

**Keywords:** Chronic hypoxia, White matter, Myelinogenesis, Neuro-function impairment, Clemastine

## Abstract

Exposure to chronic hypoxia is considered to be a risk factor for deficits in brain function in adults, but the underlying mechanisms remain largely unknown. Since active myelinogenesis persists in the adult central nervous system, here we aimed to investigate the impact of chronic hypoxia on myelination and the related functional consequences in adult mice. Using a transgenic approach to label newly-generated myelin sheaths (*NG2-CreER*^*TM*^*; Tau-mGFP*), we found that myelinogenesis was highly active in most brain regions, such as the motor cortex and corpus callosum. After exposure to hypoxia (10% oxygen) 12 h per day for 4 weeks, myelinogenesis was largely inhibited in the 4-month old brain and the mice displayed motor coordination deficits revealed by the beam-walking test. To determine the relationship between the inhibited myelination and functional impairment, we induced oligodendroglia-specific deletion of the transcription factor Olig2 by tamoxifen (*NG2-CreER*^*TM*^*; Tau-mGFP; Olig2 fl/fl*) in adult mice to mimic the decreased myelinogenesis caused by hypoxia. The deletion of Olig2 inhibited myelinogenesis and consequently impaired motor coordination, suggesting that myelinogenesis is required for motor function in adult mice. To understand whether enhancing myelination could protect brain functions against hypoxia, we treated hypoxic mice with the myelination-enhancing drug-clemastine, which resulted in enhanced myelogenesis and improved motor coordination. Taken together, our data indicate that chronic hypoxia inhibits myelinogenesis and causes functional deficits in the brain and that enhancing myelinogenesis protects brain functions against hypoxia-related deficits.

## Introduction

Chronic hypoxic exposure is well recognized as a risk factor in a number of diseases in adults, such as obstructive sleep apnea (OSA), chronic obstructive pulmonary disease (COPD), and chronic mountain sickness (CMS) [1–3]. O_2_-sensitive organs may be temporarily or permanently impaired by chronic hypoxia, and brain is one of the most sensitive organs [4]. Deficits in brain function have been noted during and after chronic hypoxic exposure, manifesting as motor abnormalities, impaired learning and memory and difficulties in attention [5–7]. Unfortunately, it remains largely unknown how chronic hypoxia causes these functional deficits in adults, and no effective therapies are available for preventing or reversing the functional deficits.

Clinical evidence has repeatedly shown by MRI imaging that chronic hypoxia can induce structural changes in the adult brain. For instance, cortical atrophy and white matter tract abnormalities have been found in CMS and OSA patients, suggesting that white matter damage may coincide with the functional deficits associated with chronic hypoxia [6, 8–10]. Myelin is an important component in CNS white matter, generated by mature oligodendrocytes (OLs). OLs project membrane-like processes and concentrically wrap axons. Myelin is not only important in insulating axons and accelerating conduction velocity, but also providing energy support to axons in the CNS [11–14]. All OLs are derived from the differentiation of oligodendrocyte precursor cells (OPCs) and this process persists throughout the lifetime [15, 16]. Myelin undergoes dynamic changes with the generation of new myelin and loss of pre-existing myelin in adults. Continuous myelin generation is important for adult brain functions, such as motor learning and spatial memory [17–21], but it remains unknown whether chronic hypoxia contributes to the inhibition of myelinogenesis in the adult brain and causes functional deficits.

In this study, we found active myelinogenesis was inhibited in the adult mouse brains by intermittent chronic hypoxia. The inhibition of new myelin generations impaired motor coordination, while the pro-myelination drug—clemastine treatment improved neurological functions in hypoxic adult mice. Together, these results indicate enhancing myelinogenesis may be a potential strategy to improve chronic hypoxia-induced functional deficits in adults.

## Materials and Methods

### Animals

Both male and female mice on a C57BL/6 background were used in all studies. All the mice were housed in a temperature- and humidity-controlled environment with free access to standard chow and water and under a 12h/12h light/dark cycle, following to the guidelines of Third Military Medical University. All mouse strains were maintained in the Third Military Medical University specific-pathogen free animal facility. The 4-month-old adult C57BL/6 mice for hypoxia models and behavioral tests were purchased from the Laboratory Animal Center of the Third Military Medical University. The reporter gene mice, *Mapt-mGFP* (*Tau-mGFP*) (The Jackson Laboratory, catalog no. 021162), have been described previously [20]. Briefly, the line was crossed with *NG2-CreER*^*TM*^ mice (The Jackson Laboratory, catalog no. 008538) to initiate recombination in OPCs. The Olig2 floxed line was crossed with the *NG2-CreER*^*TM*^*; Tau-mGFP* line to obtain *NG2-CreER*^*TM*^*; Tau-mGFP; Olig2 fl/fl* mice and heterozygous controls (*NG2-CreER*^*TM*^*; Tau-mGFP; Olig2 fl/+*). Another reporter gene mouse line, *mT/mG* (The Jackson Laboratory, catalog no. 007676), was crossed with the *PLP-CreERt* line (The Jackson Laboratory, catalog no. 005795) to obtain the *PLP-CreERt; mT/mG* line in order to label pre-existing myelin before tamoxifen induction. The genotypes of all mice were determined using PCR analysis of tail genomic DNA with appropriate primers. All animals were fed or bred in accordance with protocols approved by the Laboratory Animal Welfare and Ethics Committee of the Third Military Medical University.

### Chronic Hypoxia Model

All 4-month old animals were randomly divided into two groups. One group was placed into a hypoxia chamber (BioSpherix, ProOx P110) with the O_2_ concentration of 10%–11% (v/v) for 12 h every day. The exposure to hypoxia was carried out in the light phase and was sustained for 4 weeks [22–24]. The other group was kept in a normal O_2_ concentration (21% O_2_) in the same room, as normoxic controls.

### Administration of Tamoxifen

To induce Cre recombination in specific cell types, tamoxifen (Sigma-Aldrich, catalog no. T5648) was dissolved in sunflower oil at 30 mg/mL and administered to mice at 100 mg/kg for 4 consecutive days by oral gavage.

### Drug Treatment

Clemastine (Selleck Chemicals, Houston, TX, catalog no. S1847) was dissolved in DMSO (30 mg/mL) and diluted in the animals’ drinking water (1:20). Clemastine was finally diluted in 5% DMSO in water. The mice were treated with clemastine or an equivalent volume of vehicle (5% DMSO in water). The 4-month old hypoxic mice were given 10 mg/kg per day for 4 weeks. The mice were fed with free access to standard chow and water after drug treatment everyday.

### Tissue Processing

Each mouse was deeply anesthetized with 1% pentobarbital at 50 mg/kg and perfused with 4% paraformaldehyde in 0.1 mol/L phosphate buffer (PB; containing 0.083 mol/L Na_2_HPO_4_ and 0.017 mol/L NaH_2_PO_4_, pH 7.4) after an initial flush with 0.01 mol/L phosphate buffered saline (PBS; containing, in mmol/L, 8.3 Na_2_HPO_4_, 1.7 NaH_2_PO_4_, and 0.145 NaCl, pH 7.4). Brains were collected and postfixed with 4% paraformaldehyde in 0.1 mol/L PB overnight, then they were dehydrated in 30% sucrose in 0.01 mol/L PBS. Each brain was embedded in optimal cutting temperature compound (Sakura Finetek USA Inc., 4583) and then cut coronally at 20 μm on a cryostat microtome (CryoStar NX50, Thermo Fisher Scientific, USA). About 100 sections were collected consecutively from bregma 1.1 mm to bregma 1.34 mm. To sample in a systematic random manner, a set of 5–10 sections was sampled consecutively from each brain. In practice, every twentieth section was systematically sampled; the first was randomly selected from the first 5 sections. Floating sections were used for immunostaining.

### Immunofluorescence Staining

For immunofluorescence staining, free floating sections were blocked with 5% bovine serum albumin and 0.2% Triton-X 100 in 0.01 mol/L PBS for 2 h at room temperature, sequentially incubated with primary antibodies overnight at 4°C, and then with fluorescent-dye-conjugated secondary antibodies for 2 h at room temperature. Primary and secondary antibodies were diluted with the blocking serum buffer. The primary antibodies were: rabbit anti-NG2 (1:500, Millipore, Cat: MAB5320), rabbit anti-NF200 (1:1000, Sigma-Aldrich, Cat: N414), rat anti-MBP (1:200, Millipore, Cat: MAB386), rabbit anti-NeuN (1:1000, Abcam, Cat: ab177487) and rat anti-CD31 (1:200, BD Biosciences, Cat: 553370). The secondary antibodies were: AlexaFluor-488-, AlexaFluor-568-, or AlexaFluor-647-conjugated antibodies against goat, rabbit or rat (1:1000; Life Technologies). Nuclei were counterstained with DAPI.

### Image Acquisition and Quantification

Fluorescence images were captured using a confocal laser-scanning microscope (Olympus, FV3000) with excitation wavelengths appropriate for AlexaFluor-488 (488 nm), -596 (568 nm), -647 (628 nm), or DAPI (380 nm). The corpus callosum, striatum, anterior commissure (AC), hippocampus (CA1 and fimbria), motor cortex (M1 and M2), and sensory cortex (S2) were selected from each sample for quantification. The reporter gene mouse line (*NG2-CreER*^*TM*^*; Tau-mGFP*) was used to label newly-formed myelin after tamoxifen induction. mGFP-positive density was calculated using Image-Pro Plus 5.0 (Media Cybernetics).

### Behavioral Tests

All mice were fed in a controlled environment (25°C) with free access to food and water and housed under a 12 h/12 h day/night cycle. All tests were performed between 12:00 and 18:00. After each trial, all apparatus was wiped with 30% ethanol to avoid odor cues. In all behavioral experiments, investigators were blinded to genotype and mice were handled gently to avoid stress.

#### Open Field Test

Open field tests were performed in an open-field apparatus (Biowill, Shanghai, China). Briefly, each mouse was placed in the center of the open-field box (50 × 50 × 50 cm^3^), then its activity was recorded for 5 min. The total distance traveled was measured to assess general motor function.

#### Beam Walking Test

A modified beam walking test was used to assess the motor coordination of mice. In the test, a beam (0.4 cm wide) was placed 50 cm above the floor in a dark room, with one end illuminated by a lamp and the other end in a non-transparent box (20 × 20 × 20 cm^3^). Each mouse was placed at the end with light and trained three times per day for 3 days before testing. In order to attract the mouse to walk across the beam in a sequence of 30-, 50- and 70-cm distances, padding material from its cage was placed in the box during the training days. During the test, videos were collected from both sides of the beam to record the performance. The frequency of foot-slips when the mouse walked a distance of 80 cm was calculated as the index of beam walking performance [25].

### Statistical Analysis

Mice were randomly assigned to experimental time points or groups, and no animals or data points were excluded from analyses. Investigators were blinded to group allocation during data collection and analysis. All data are presented as the mean ± SEM. Points represent individual animals. Statistical difference between groups was determined using the two-tailed unpaired *t*-test or one-way ANOVA followed by *post hoc* Tukey’s test. The statistical methods, exact *n* values, significant and non-significant *P* values, and degrees of freedom are described in the figure legends. Significance is reported as *P < 0.05, **P < 0.01, or ***P < 0.001.

## Results

### Active Myelinogenesis in Adult Mouse Brain

**Fig. 1:**
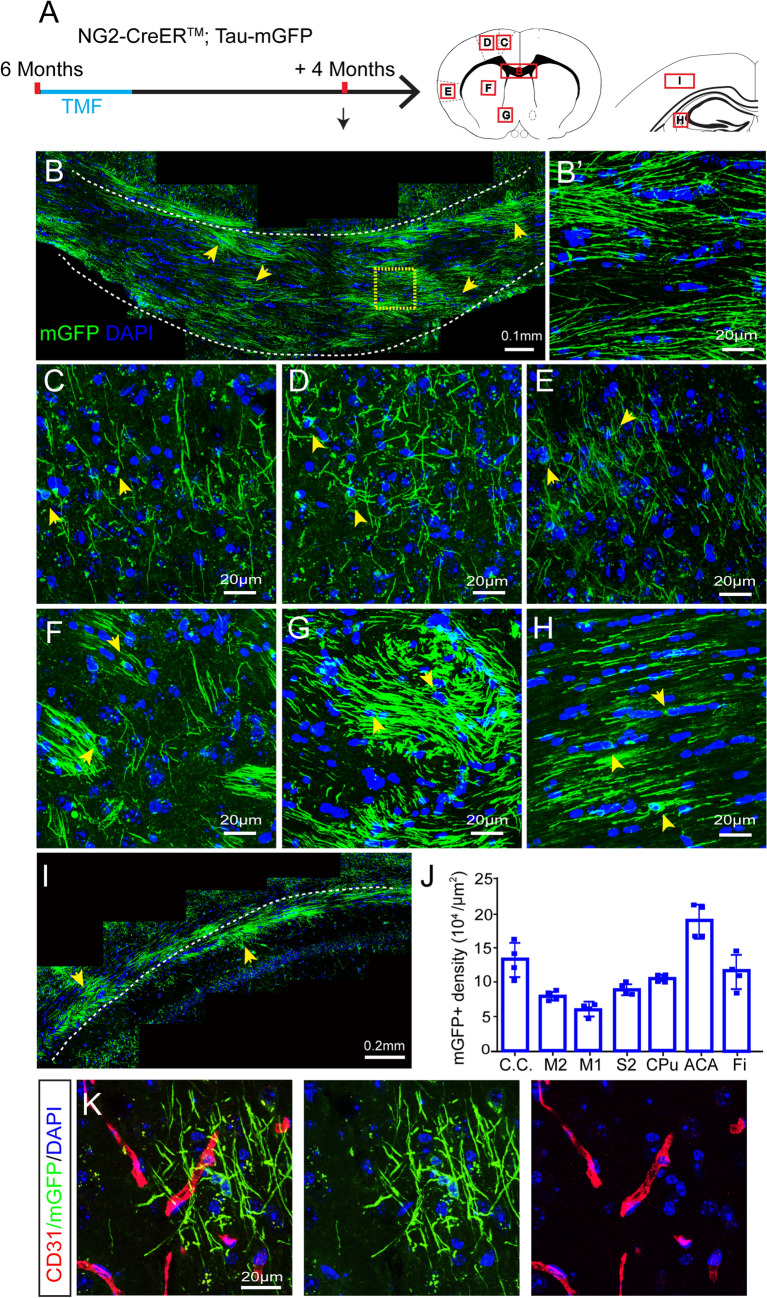
New myelin generation in adult mouse brain. **A** Schematic diagram displaying the time-course of tamoxifen induction and histology in *NG2-CreER*^*TM*^*; Tau-mGFP* mice (letters refer to panels **B**–**I** below). **B** Representative immunofluorescence image displaying mGFP-positive oligodendrocytes and myelin sheaths (yellow arrows) in the corpus callosum. **B’** Enlargement of boxed area in **B**. **C–I** Representative immunofluorescence images displaying mGFP-positive oligodendrocytes and myelin sheaths (yellow arrows) in M2 (**C**), M1 (**D**), S2 (**E**), CPu (**F**), AC (**G**), Fi (**H**), and CA1 (**I**). **J** Quantification of mGFP-positive de nsity in the CC, M2, M1, S2, CPu, AC, and Fi (points represent individual animals; *F* = 22.40, *P* < 0.001, one-way ANOVA followed by the *post hoc* Turkey test, error bars represent the mean ± SEM). **K** Representative immunofluorescence images showing mGFP-positive areas and CD31-positive vessels in the cortex. TMF, tamoxifen; CC, corpus callosum; M2, secondary motor cortex; M1, primary motor cortex; S2, secondary somatosensory cortex; CPu, caudate putamen striatum; AC, anterior commissure; Fi, fimbria (hippocampus).

We set out to map myelinogenesis in the adult brain by using *NG2-CreER*^*TM*^*; Tau-mGFP* transgenic mice. Upon recombination, newly-generated myelin sheaths and their associated OLs start to express mGFP [20, 26]. Recombination was induced in the transgenic mice at the age of 6 months and mGFP-positive myelin and mature OLs were observed in different regions of the whole brain 4 months after induction (Fig. 1A), including the corpus callosum (Fig. 1B, B’), M2 and M1 (Fig. 1C and D), sensory cortex (Fig. 1E), caudate putamen striatum (CPu) (Fig. 1F), anterior commissure (AC) (Fig. 1G), and hippocampus (Fig. 1H and I). We calculated the density of newly-formed mGFP-positive myelin, and found that more myelin sheaths were generated in the AC and corpus callosum of adult mice (Fig. 1J). These results indicate that myelinogenesis is highly active in all brain regions in adult mice. As pericytes are closely associated with vascular vessels in the brain and also express NG2, we investigated whether tau-mGFP is expressed in pericytes by immunostaining for CD31, a marker for vascular epithelial cells. We did not detect Tau-mGFP positive signal on and around CD31 positive vessels (Fig. 1K), suggesting that pericytes do not express mGFP in the *NG2-CreER*^*TM*^*; Tau-mGFP* mouse.

### Chronic Hypoxia Inhibits Myelinogenesis in Adult Mice

**Fig. 2:**
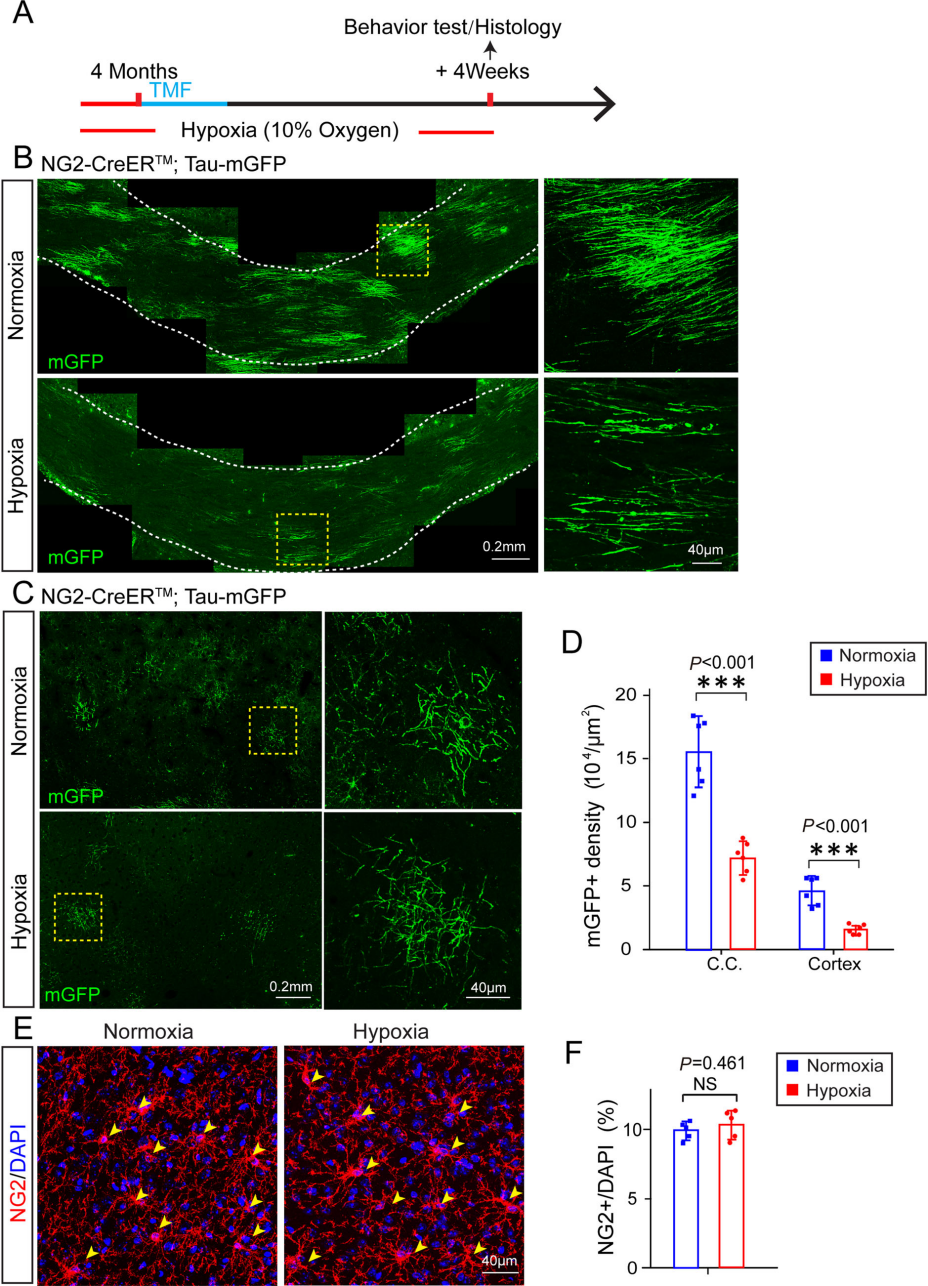
Chronic hypoxia inhibits myelinogenesis in adult mice. **A** Schematic diagram of the time-course of hypoxic exposure, tamoxifen induction, histology and behavioral testing. **B, C** Left, representative immunofluorescence images displaying mGFP-positive oligodendrocytes and myelin sheaths in the corpus callosum (**B**) and cortex (**C**). Right, enlargements of the boxed areas in the left panels. **D** Quantification of mGFP-positive density in the corpus callosum and cortex of normoxic and hypoxic adult mice (*n* = 6 biologically independent mice per group; corpus callosum: *t*_(10)_ = 6.798, *P* < 0.001; cortex: *t*_(10)_ = 6.254, *P* < 0.001; two-tailed unpaired *t*-test). **E** Immunostaining for NG2 displaying OPCs (yellow arrowheads)in hypoxic and normoxic brains. **F** Percentages of NG2-positive cells among DAPI-positive cells in normoxic and hypoxic brains (*n* = 5 biologically independent mice per group; *t*_(8)_ = 0.773, *P* = 0.461, two-tailed unpaired *t*-test). Points represent individual animals. Error bars represent the mean ± SEM. NS, not significant; ***P < 0.001.

To explore the influence of chronic hypoxia on myelinogenesis in adult mice, we exposed 4-month old *NG2-CreER*^*TM*^*; Tau-mGFP* mice to 10% O_2_ (v/v) for 12 h per day. The mice were placed in a hypoxia chamber for 4-day pre-conditioning before tamoxifen induction, then the mice were randomly divided into two groups for hypoxic or normoxic exposure (Fig. 2A). After 4-weeks of exposure to hypoxia, the brains were collected for assessment of mGFP-positive new myelin. It was evident that the mGFP-positive new myelin and OLs were greatly decreased in the corpus callosum and motor cortex of the hypoxic mice compared to the normoxic controls (Fig. 2B-D). Given that myelination depends on the differentiation and maturation of OPCs, we then assessed the OPC numbers by immunostaining for NG2 to explore whether the decreased myelination induced by chronic hypoxia involved the OPC population (Fig. 2E). We calculated the ratio of NG2-positive cells to DAPI-positive nuclei and found that the percentage of NG2-positive cells did not significantly change (Fig. 2F), suggesting that chronic hypoxia inhibits OPC differentiation and myelination, rather than altering the OPC population in adult mice.

### Chronic Hypoxia Does not Cause Significant Myelin Degeneration

**Fig. 3:**
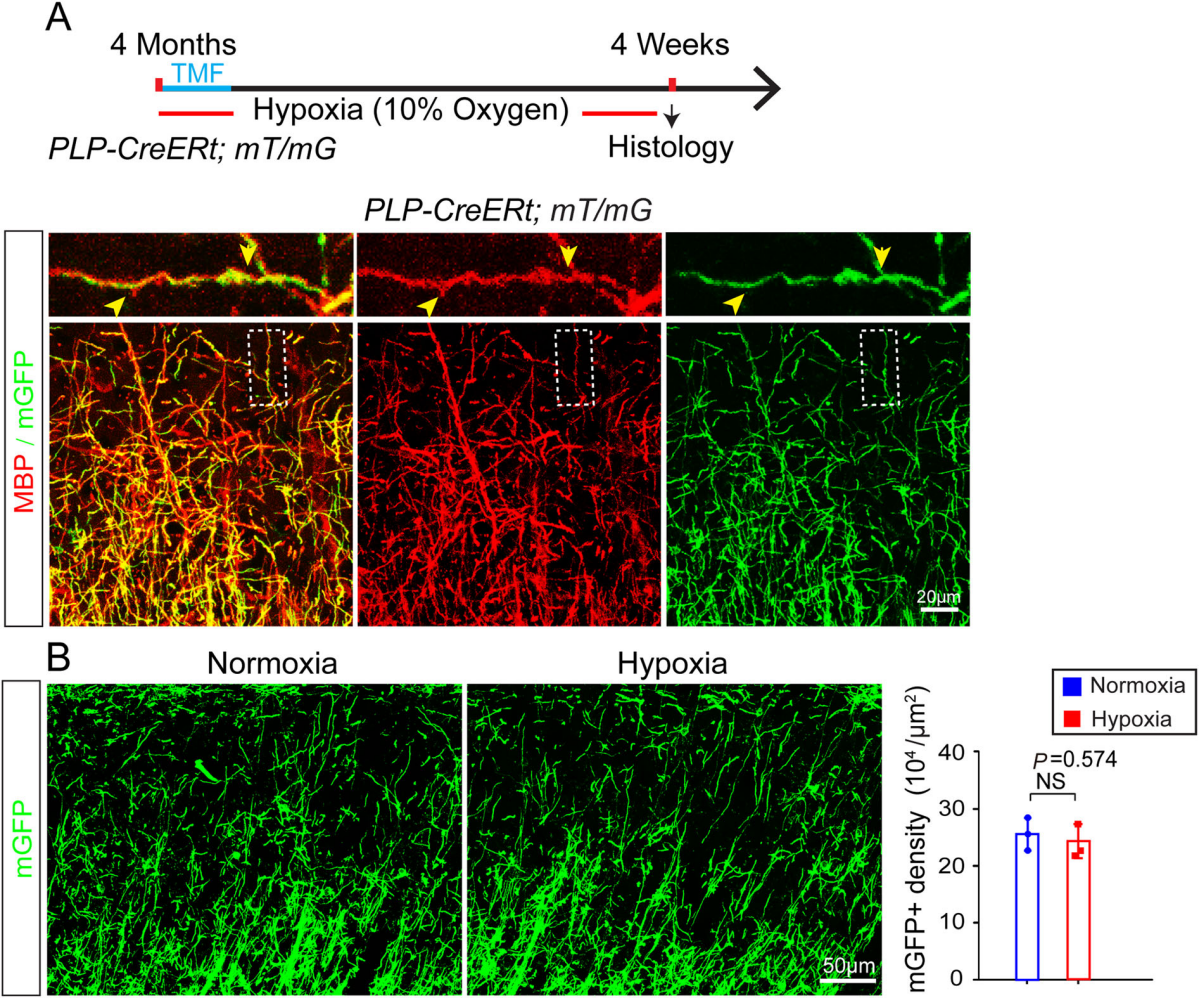
Pre-existing myelin sheaths in the hypoxic adult brain. **A** Upper panel, schematic diagram of the time-course of hypoxic exposure, tamoxifen induction, and histology. Lower panels, representative images showing mGFP-positive areas co-localized with MBP-positive myelin sheaths in the cortex of *PLP-CreERt; mT/mG* mice, 4 weeks after recombination at 4 months. **B** Representative images and quantification of mGFP-positive density in the cortex of normoxic and hypoxic *PLP-CreERt; mT/mG* adult mice (*n* = 3 biologically independent mice per group; *t*_(4)_ = 0.611, *P* = 0.574, two-tailed unpaired *t*-test). Points represent individual animals. Error bars represent the mean ± SEM. NS, not significant.

To understand whether pre-existing myelin in the brain is sensitive to hypoxia, we used the *PLP-CreERt; mT/mG* line to label pre-existing myelin [20]. The recombination driven by the *plp* gene only occurs in mature OLs and their associated myelin sheaths but not in OPCs, allowing for tracing the fate of pre-existing myelin sheaths. Recombination was induced for 4 days before the mice were placed in the hypoxic chamber at the age of 4 months. The mGFP-positive myelin sheaths exclusively co-expressed myelin basic protein (MBP) as revealed by MBP immunostaining (Fig. 3A). We examined the *PLP-CreERt; mT/mG* brains after 4 weeks of exposure to hypoxia and quantified the mGFP-positive myelin sheaths in the superficial layers of cortex, where myelin is comparatively sparse. Quantification did not reveal a significant change of mGFP-positive myelin in the hypoxic brain as compared to normoxic controls (Fig. 3B), suggesting that the pre-exiting myelin is stable in response to hypoxic insult.

### Chronic Hypoxia Impairs Neuronal Functions in Adult Mice

**Fig. 4:**
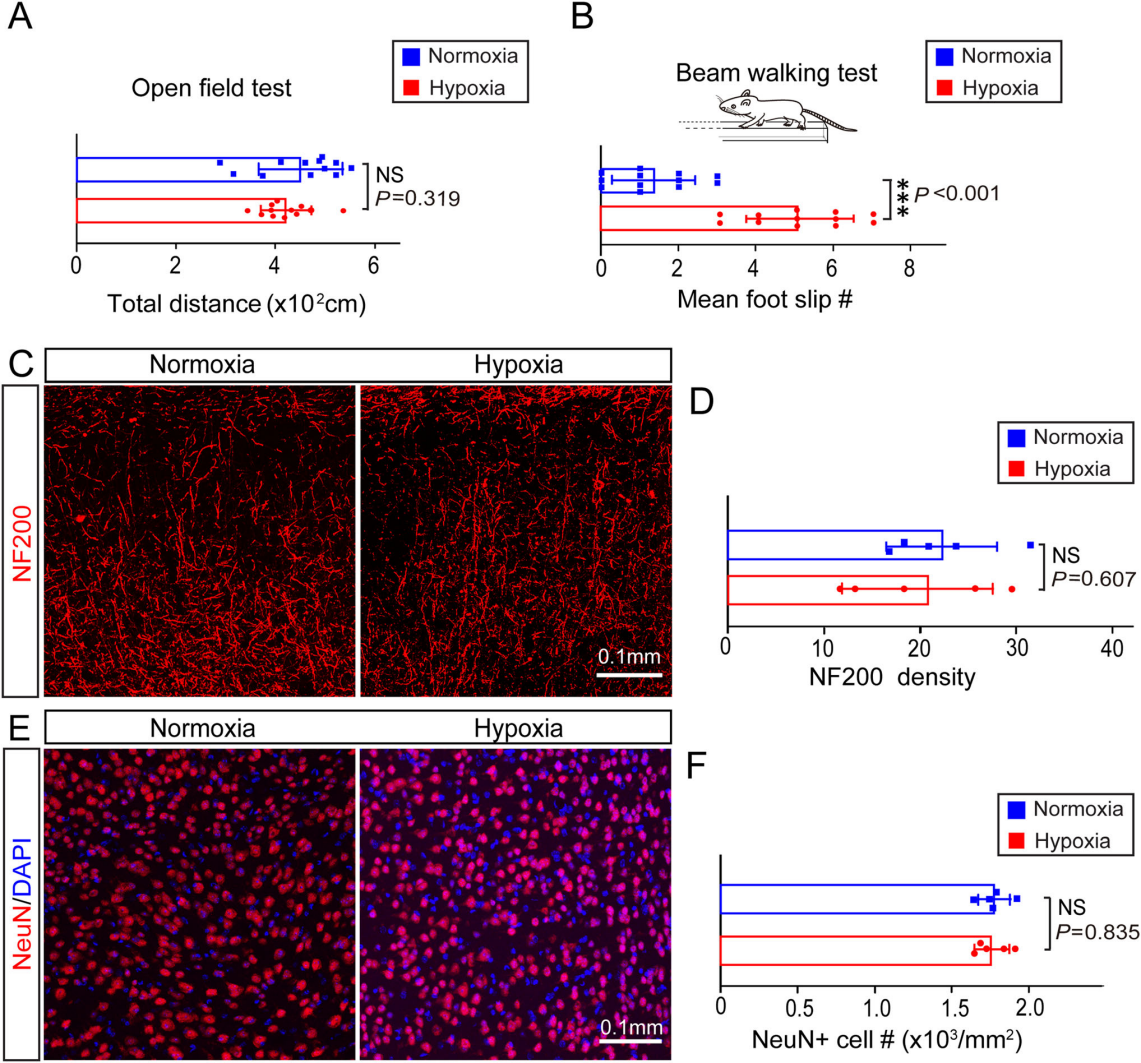
Chronic hypoxia causes motor deficits in adult mice. **A** Total distance of hypoxic and normoxic mice in the open field test (*n* = 12 biologically independent mice per group; *t*_(22)_ = 1.019, *P* = 0.319, two-tailed unpaired *t*-test). **B** Mean number of foot-slips of hypoxic and normoxic mice in the beam-walking test (*n* = 12 biologically independent mice per group; *t*_(22)_ = 7.435, *P* < 0.001, two-tailed unpaired t-test). **C** Representative immunofluorescence images displaying NF200 expression in normoxic and hypoxic brains. **D** Quantification of NF200 density in normoxic and hypoxic brains (*n* = 5 biologically independent mice per group; *t*_(8)_ = 0.607, *P* = 0.535, two-tailed unpaired *t*-test). **E** Representative immunofluorescence images showing NeuN-positive (red) cells in normoxic and hypoxic cortex. **F** Quantification of NeuN-positive cells in normoxic and hypoxic brains (*n* = 5 biologically independent mice per group; *t*_(8)_ = 0.215, *P* = 0.835, two-tailed unpaired *t*-test). Points represent individual animals. Error bars represent the mean ± SEM. NS, not significant; ***P < 0.001.

We next assessed whether chronic hypoxia caused functional deficits in adult mice. Open field test showed no significant difference in total distance travelled between normoxic and hypoxic mice (Fig. 4A), indicating that chronic hypoxia does not impair general activity in adults. Then we applied the modified beam-walking test to assess motor coordination in mice after chronic hypoxia [25]. We counted the total number of slips of the hind-limbs during performance of the task. The total number of slips by the hypoxic group was greater than those in the wild-type controls (Fig. 4B), suggesting that chronic hypoxia impairs neuronal function in adult mice. To determine if this functional deficit is associated with neuronal loss and axon degeneration, we immunostained for NeuN and NF200, respectively. Our results showed that the densities of NF200-positive axons and NeuN-positive neurons in chronic hypoxic brains did not significantly differ from normoxic controls (Fig. 4C-F). These results indicate that the impairments caused by chronic hypoxia unlikely to be due to significant neuronal loss or axon degeneration.

**Fig. 5:**
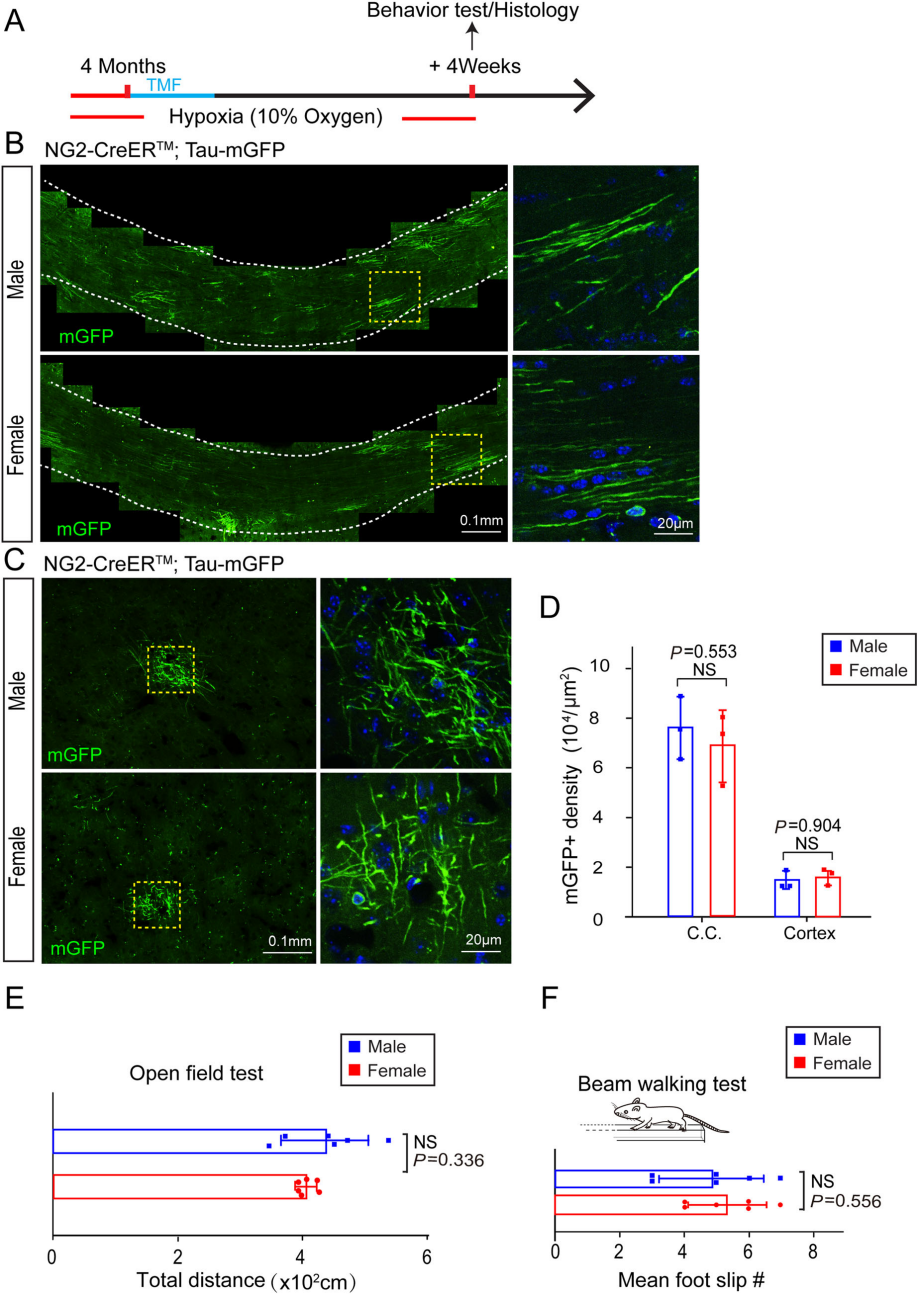
Hypoxic adult mice show no gender difference in myelinogenesis and functional deficits. **A** Schematic diagram of the time-course of hypoxic exposure, tamoxifen induction, histology and behavioral tests. **B, C** Left panels, representative immunofluorescence images displaying mGFP-positive oligodendrocytes and myelin sheaths in the corpus callosum (**B**) and cortex (**C**). Right panels, enlargements of the boxed areas in the left panels. **D** Quantification of mGFP-positive density in the corpus callosum and cortex of normoxic and hypoxic adult mice (*n* = 3 biologically independent mice per group; corpus callosum (C.C.): *t*_(4)_ = 0.646, *P* = 0.553; cortex: *t*_(4)_ = 0.128, *P* = 0.903; two-tailed unpaired *t*-test). **E** Total distance of male and female mice in the open field test (*n* = 6 biologically independent mice per group; *t*_(10)_ = 1.009, *P* = 0.336; two-tailed unpaired *t*-test). **F** Mean number of foot-slips of hypoxic and normoxic mice in the beam-walking test (*n* = 6 biologically independent mice per group; *t*_(10)_ = 0.609, *P* = 0.556; two-tailed unpaired *t*-test). Points represent individual animals. Error bars represent the mean ± SEM. NS, not significant.

To explore whether gender differences play a role in the hypoxia-induced changes in myelinogenesis and functional injury, we exposed age-matched male and female mice to 10% O_2_ (v/v) for 12 h per day. After 4 weeks of exposure to hypoxia, we carried out behavioral tests and the brains were collected for assessment of mGFP-positive new myelin (Fig. 5A). We found that the densities of new myelin and OLs in the cortex and corpus callosum did not significantly differ between male and female mice (Fig. 5B-D). Consistent with this, neither the open field test nor the modified beam-walking test revealed significant differences between male and female mice (Fig. 5E, F). These results suggest that gender differences do not bias myelinogenesis and neuronal function in mice exposed to chronic hypoxia.

### Inhibited Myelinogenesis Impairs Neuronal function in Adults

**Fig. 6:**
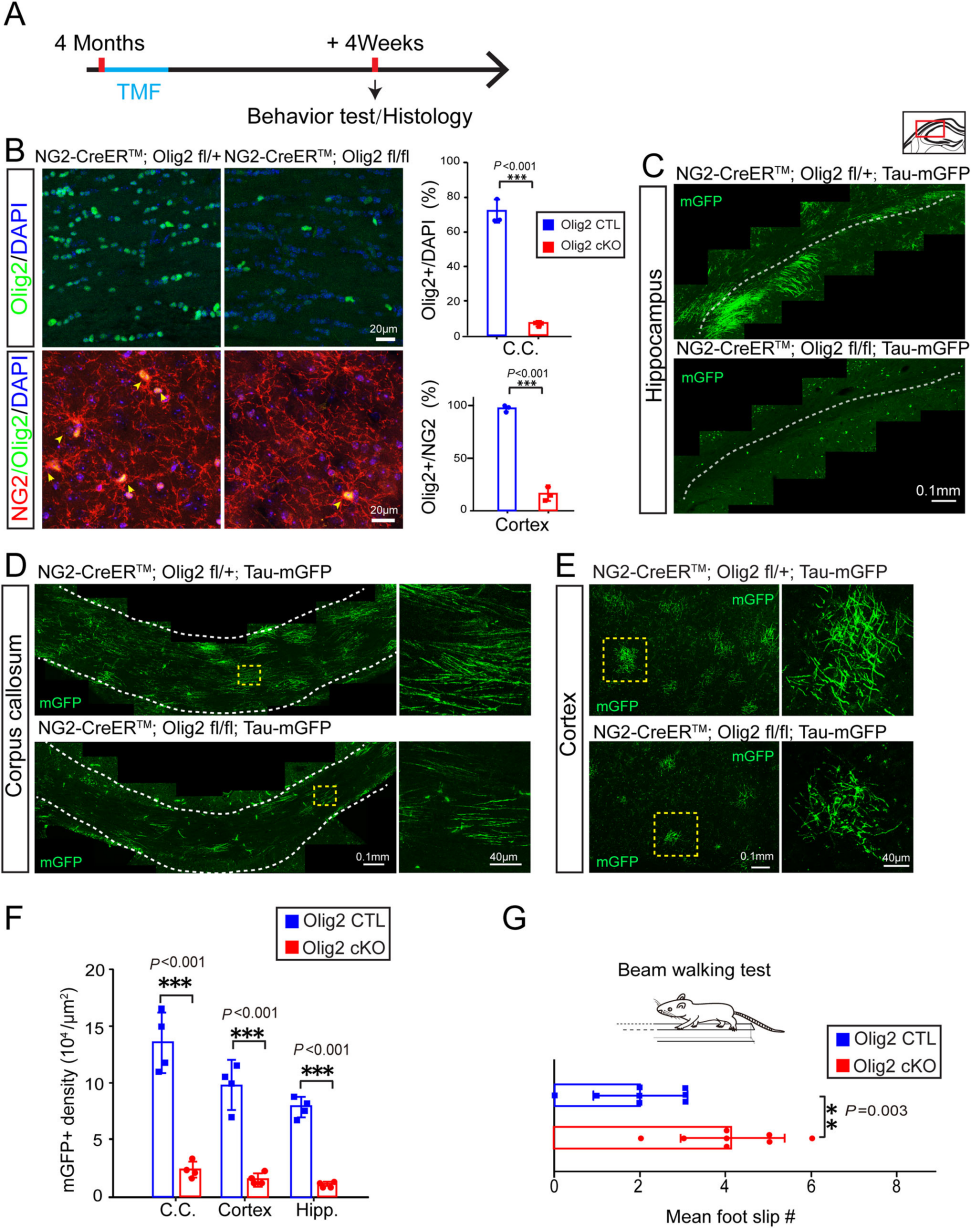
Olig2 deletion in OPCs inhibits myelination and impairs motor function in adult mice**. A** Schematic diagram of the time-course of tamoxifen induction, histology and behavioral testing. **B** Upper panels, representative immunofluorescence images of Olig2 (red) counterstained with DAPI (blue) (left and middle panels) and quantification of the percentage of Olig2-positive cells among DAPI-positive cells (right panel) in corpus collasum (C. C.) of Olig2-cKO brains and the heterozygous controls. Lower panels, Olig2 (red) and NG2 (green) counterstained with DAPI (blue) (left and middle panels) and quantification of the percentage of Olig2-positive cells among NG2-positive cells in cortex (right panel) of Olig2-cKO brains and the heterozygous controls (*n* = 3 biologically independent mice per group; corpus callosum: *t*_(4)_ = 17.08, *P* < 0.001; cortex: *t*_(4)_ = 10.07, *P* <0.001; two-tailed unpaired *t*-test). **C-E** Representative images showing mGFP-positive new myelin in the hippocampus (**C**), corpus callosum (**D**), and cortex (**E**) of Olig2-cKO brains and heterozygous controls (right panels in **D** and **E** show enlargements of boxed areas). **F** Quantification of mGFP-positive density in Olig2-cKO brains and controls (*n* = 4 biologically independent mice per group; corpus callosum (C.C.): *t*_(6)_ = 8.252, *P* < 0.001; cortex: *t*_(6)_ = 7.371, *P* < 0.001; hippocampus (Hipp.): *t*_(6)_ = 15.51, *P* < 0.001; two-tailed unpaired *t*-test). **G** Mean number of foot-slips of Olig2-cKO mice and age-matched controls in the beam-walking test (*n* = 8 biologically independent mice per group; *t*_(14)_ = 3.660, *P* = 0.003, two-tailed unpaired *t*-test). Points represent individual animals. Error bars represent the mean ± SEM. **P < 0.01; ***P < 0.001.

Since hypoxia induced functional deficits and inhibited myelogenesis in adult mice, we next examined whether there is a causal relationship between inhibited myelinogenesis and the deficits in motor coordination. We chose to conditionally delete Olig2 in OPCs so as to inhibit myelinogenesis without affecting other cell types, as Olig2 is a transcriptional factor that is specifically expressed by oligodendroglial lineage cells and is important in regulating OPCs differentiation and myelination [27, 28]. To that end, we crossed the reporter mouse line *NG2-CreER*^*TM*^*; Tau-mGFP* with the Olig2 floxed line to obtain Olig2 conditional knockout (cKO) mice (*NG2-CreER*^*TM*^*; Tau-mGFP; Olig2 fl/fl*) and heterozygous controls (*NG2-CreER*^*TM*^*; Tau-mGFP; Olig2 fl/+*). Tamoxifen was administered at 4 months and the mice were sacrificed 4 weeks later (Fig. 6A). We detected Olig2 expression after induction by immunostaining for Olig2 and our results indicated that the number of Olig2-positive cells was decreased greatly in the corpus callosum (Fig. 6B). Using double immunostaining for Olig2 and NG2, we also found that Olig2 expression was almost absent in NG2-positive OPCs (Fig. 5B), indicating efficient Olig2 deletion in the Olig2-cKO brain. As expected, much less newly-formed myelin (mGFP-positive) was observed in the corpus callosum, hippocampus, and motor cortex of Olig2-cKO mice than in heterozygous controls, suggesting that Olig2 deletion in OPCs mimics the decreased myelinogenesis induced by chronic hypoxia (Fig. 6C-F). Again, we examined the motor coordination of Olig2-cKO mice using the beam-walking test. The results showed that there were significantly more foot slips in adult Olig2-cKO mice than that in heterozygous controls (Fig. 6G), suggesting that myelinogenesis is necessary for motor coordination in adults.

### Enhancing Myelination with Clemastine Prevents Hypoxia-induced Functional Deficits

**Fig. 7:**
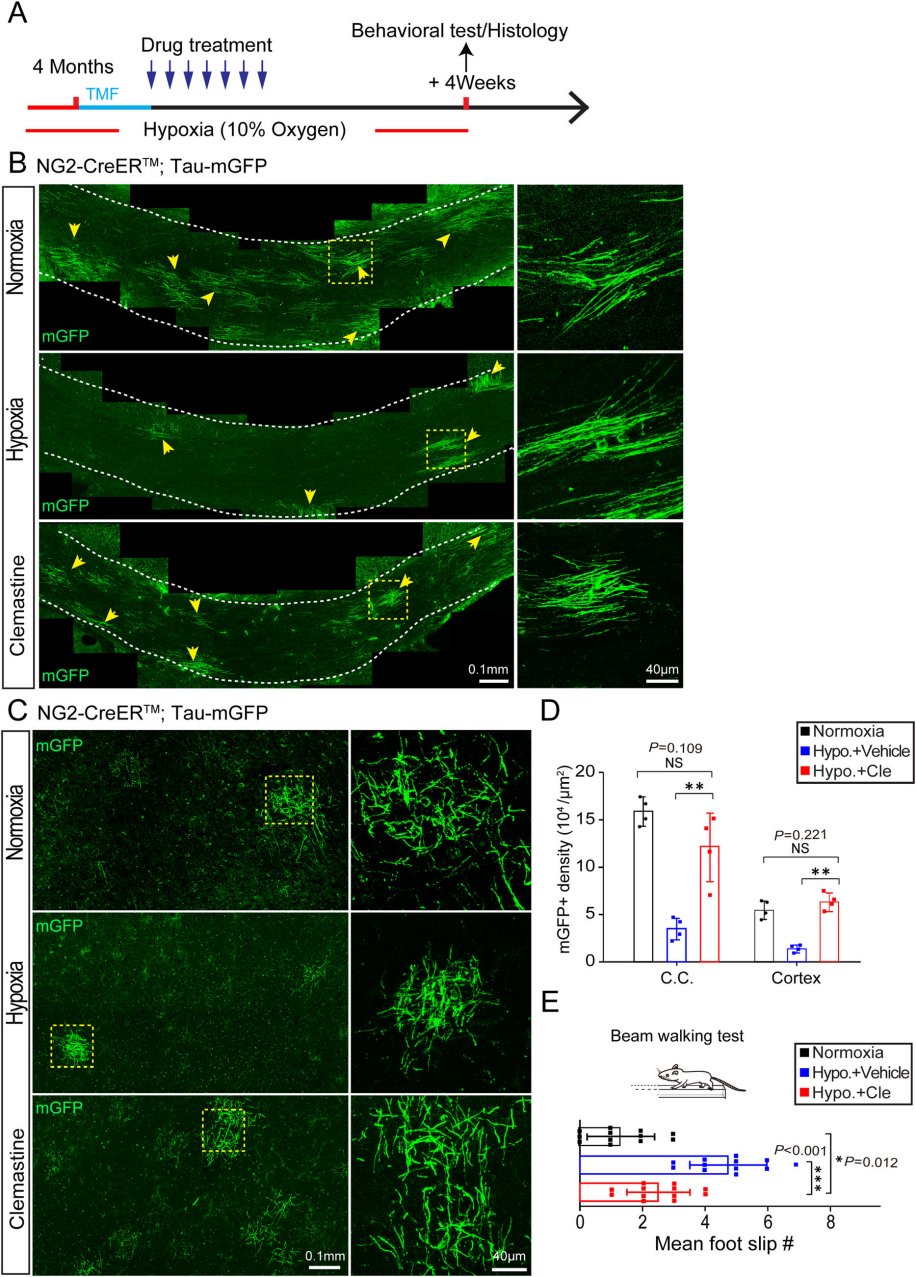
Clemastine treatment during hypoxia rescues the functional impairment. **A** Schematic diagram of the time-course of tamoxifen induction, hypoxic exposure, drug treatment, histology and behavioral test. **B–D** Representative images and quantification (**D**) of mGFP-positive new myelin (arrowheads) in the corpus callosum (**B**) and cortex (**C**) of normoxic control, hypoxic vehicle-treated, and hypoxic clemastine-treated adult mice (right panels in **B** and **C** show enlargements of the boxed areas in the left panels) (*n* = 4 biologically independent mice per group; corpus callosum: *t*_(6)_ = 4.605, *P* < 0.01 (hypoxic vehicle-treated *vs* hypoxic clemastine-treated). *t*_(6)_ = 1.879, *P* = 0.109 (normoxic control *vs* hypoxic clemastine-treated); cortex: *t*_(6)_ = 9.487, *P* < 0.001 (hypoxic vehicle-treated *vs* hypoxic clemastine-treated). *t*_(6)_ = 1.363, *P* = 0.221 (normoxic control *vs* hypoxic clemastine-treated); two-tailed unpaired *t*-test). **E** Mean number of foot-slips of normoxic control, hypoxic vehicle-treated and hypoxic clemastine-treated adult mice in the beam-walking test (*n* = 12 biologically independent mice per group; *t*_(22)_ = 4.952, *P* < 0.001 (hypoxic vehicle-treated *vs* hypoxic clemastine-treated). *t*_(22)_ = 2.755, *P* = 0.012 (normoxic control *vs* hypoxic clemastine-treated); two-tailed unpaired *t*-test). Points represent individual animals. Error bars represent the mean ± SEM. NS, not significant; **P < 0.01; ***P < 0.001.

Since myelinogenesis is necessary for motor coordination in adults, we next explored whether enhancing OL differentiation in adults improves the functional deficits associated with chronic hypoxia. Clemastine is an FDA-approved muscarinic receptor antagonist that is effective in improving myelinogenesis *in vivo* [29, 30]. Recombination was induced by tamoxifen treatment before hypoxia exposure. Here, we gave clemastine to 4-month-old reporter gene mice (*NG2-CreER*^*TM*^*; Tau-mGFP*) for 4 weeks, while they were exposed to chronic hypoxia (Fig. 7A). Strikingly, we found that clemastine treatment greatly increased the mGFP-positive newly-formed myelin in the motor cortex and corpus callosum (Fig. 7B-D), and the density of new myelin recovered to the same levels as in the normoxic controls, indicating that this drug enhances myelinogenesis in brains exposed to chronic hypoxia. Next, we applied the beam-walking test, and found that clemastine-treated mice showed fewer foot-slips than hypoxic wild-type controls, but remained significantly more than that in normoxic mice, indicating that pathological changes other than myelinogenesis are also involved in the hypoxia-related deficits (Fig. 7E). These results suggest that myelination-enhancing therapeutics may be a promising strategy to prevent hypoxia-induced impairments of neuronal function in adults.

## Discussion

Chronic hypoxia is a detrimental condition that may impair organ functions and the CNS is remarkably susceptible to insufficiency of the O_2_ supply. For instance, exposure of the developing CNS to hypoxia may result in long-term functional deficits. Hypoxia-related white matter injury in preterm infants can lead to long-term cognitive and motor deficits in childhood and adulthood [31–35]. It is noteworthy that adults respond differently in tolerating hypoxia; for example, the CNS is remarkably more sensitive in adults than in neonates. Clinical studies have demonstrated that adult OSA and CMS patients display long-term deficits, including motor and cognitive functions [2, 6, 7, 36]. Ten percent O_2_ is widely used to mimic chronic hypoxia in adult and neonatal rodent models. Here, we used 10% O_2_ to investigate a hypoxic insult in adult animals. Admittedly, it is unclear how the environmental O_2_ affected oxyhemoglobin saturation in this case; nevertheless this hypoxic exposure causes pathological changes in the CNS [37–39]. Our results showed that 4 weeks of hypoxia disrupted motor coordination, which is in line with hypoxia-related disruption of neuronal function in adulthood. It is noteworthy that hypoxic exposure can cause extensive brain dysfunction, such as cognitive decline and memory deficits in humans and rodents [40, 41]. It is well-known that chronic hypoxia impairs other organs including the heart, lungs and muscles [3, 42]. Thus, it is possible that dysfunctions in motor coordination may not be solely due to inhibited myelinogenesis. Given that inhibited myelinogenesis by Olig2 deletion in OPCs also caused motor dysfunctions in Olig2-cKO mice, myelinogenesis plays a crucial role in the impaired motor function in response to chronic hypoxia.

It remains unclear how hypoxia causes functional deficits in adult mice. White matter abnormalities have been reported in OSA and CMS patients [8, 43–46]. As an important component of the CNS, myelin is highly enriched in the white matter and abundant in the gray matter. Myelin undergoes dynamic changes with the generation of new myelin and degeneration of pre-existing myelin in the adult CNS. Recent evidence has shown that myelinogenesis persists throughout the lifetime [47, 48], but it is difficult to distinguish newly-formed myelin from pre-existing myelin in adults by conventional immunostaining. Here, we used a transgenic reporter mouse model that labeled newly-formed myelin and found that myelinogenesis was highly active in most brain regions, suggesting that it is important for sustaining neuronal functions. We did not detect any positive signal in OPCs and pericytes (NG2-positive cells) in the *NG2-CreER*^*TM*^*,* *Tau-mGFP* brain, probably due to the insertion of the *mGFP* gene sequence is after the *tau* promoter, and these cells might barely express tau. More importantly, our results indicate that chronic hypoxia inhibits myelinogenesis in the adult CNS but is unlikely to induce neuronal loss or axon degeneration [26, 49, 50]. Compared to the normoxic group, we also found that the NG2-positive cell density did not significantly change in the hypoxic brain, suggesting that the OPC population is not sensitive to hypoxia. It has been repeatedly demonstrated that adult OPCs proliferate to substitute for those undergoing differentiation and maintain the population in adult and aging brains [20, 51, 52]. We speculate that OPC differentiation is inhibited by hypoxia, so that much less mature OLs are generated in the brain.

It is interesting that 10% O_2_ exposure did not cause significant neuronal loss or axon degeneration, as previous studies have demonstrated that chronic hypoxia inhibits neurogenesis in the dentate gyrus in adults [37–39]. Damage to neurons and other cell types remains possible, since the enhancement of myelinogenesis did not result in complete functional recovery. It is evident that the functional impairments in hypoxic adults can be caused largely by decreased myelinogenesis. Notably, recent evidence has shown that that myelinogenesis in adulthood is required for spatial memory capacity and consolidation, as well as working memory and learning [20, 53, 54]. By using Olig2-cKO mice, we found that the inhibition of myelinogenesis by Olig2 knockout in OPCs impaired motor coordination, demonstrating that inhibited myelinogenesis can directly cause functional deficits in adulthood. Consistent with this, Olig2 deficiency during development also leads to myelin deficits and cognitive dysfunction [55]. The exact mechanisms underlying the behavioral outcomes remain unclear. It is well known that myelin sheaths insulate and nourish axons and that newly-added myelin sheaths may change the activity and gene expression of neurons, so as to facilitate the establishment of neural circuitry [56–58]. Thus, it is plausible that the change in myelin substantially affected neuronal functions and this ultimately altered the behavioral outcome in hypoxic mice.

Currently, no effective therapies are available for the treatment of the neuro-functional deficits in patients with hypoxic insults. Regarding the evidence that chronic hypoxia inhibits myelinogenesis and consequently disrupts neuronal functions, enhancing myelination presents a promising approach to rescuing the deficits. Here, we showed that clemastine treatment in adult mice rescued the deficits induced by chronic hypoxia. In support of this notion, clemastine also rescues the myelin damage and improves the behavioral alterations in a mouse model of Williams syndrome, and prevents the decline in memory capacity in aged mice [20, 56]. Clemastine can pass through the blood brain barrier and improved myelination and neuronal function in a clinical trial of multiple sclerosis [59]. It is not completely clear in this case that clemastine enhances myelination solely by blocking muscarinic receptor 1 (M1R), given its binding affinity to a wide range of receptors, such as histamine receptors and other muscarinic receptor subtypes [60], and it was not clear that clemastine could regulate oxygen level or Olig2 expression directly. Considering the side-effects of clemastine, ataxia and impaired coordination [61], it is necessary to develop M1R specific myelin-enhancing drugs with minimal side-effects. Nevertheless, the strategy of enhancing myelination may be of potential value in treating hypoxia-associated dysfunctions in adults.
